# Comparative analysis of microbial community structure in different times of *Panax ginseng* Rhizosphere microbiome and soil properties under larch forest

**DOI:** 10.1186/s12863-023-01154-1

**Published:** 2023-09-14

**Authors:** Tong Aizi, Liu Lijuan, Liu Lihua, Liu Wei, Qin Jiamei

**Affiliations:** Key Laboratory of Evaluation and Application of Changbai Mountain Biological Germplasm Resources of Jilin Province, College of Life Science, Tonghua Normal University, Tonghua, 134002 China

**Keywords:** Larch forest, Forest ginseng, Rhizosphere microorganisms, Diversity, Community composition

## Abstract

**Background:**

Panax ginseng cultivated under the forest is popular because its shape and effective ingredients are similar to wild ginseng. The growth of P. ginseng in the larch forest is generally better than in the broad-leaved forest, and the incidence rate of diseases is low. Therefore, the selection of forest species is one of the basic factors in the successful cropping of P. ginseng.

**Methods:**

Illumina HiSeq high-throughput sequencing was used to analyze the 16S rRNA/ITS gene sequence of P. ginseng rhizosphere soil under larch forest to study the rhizosphere microbiome's diversity and community composition structure.

**Results:**

The species classification and richness of rhizosphere bacterial and fungal communities in the same-aged P. ginseng were similar. Consistent with the soil system of commonly cultivated crops, Proteobacteria, Actinobacteriota, Acidobacteriota, Verrucomicrobiota, Chloroflexi, and Basidiomycota, Ascomycota were the dominant phylum of bacteria and fungi, respectively. Compared with the soil without planting P. ginseng, the diversity of microorganisms and community structure of continuous planting for 2 years, 5 years, and 18 years of *P. ginseng* rhizosphere soil had little change. The accumulation levels of Ilyonectria, Fusarium, Gibberella, and Cylindrocarpon were not significantly increased with planting P. ginseng and the increased age of cropping *P. ginseng.*

**Conclusions:**

The results of this study showed that the soil function of the larch forest was good, which provided a theoretical basis for the land selection and soil improvement of cultivating *P. ginseng* under the larch forest.

**Supplementary Information:**

The online version contains supplementary material available at 10.1186/s12863-023-01154-1.

## Background

Ginseng (*Panax ginseng* C. A. Mey.), which belongs to the Araliaceae family *Panax* genus, is a traditional precious Chinese herbal medicine with important economic value [1]. The cultivation mode can be divided into the cultivation of *P. ginseng* under the forest, the cultivation of *P. ginseng* in deforestation, and the cultivation of *P. ginseng* in the farmland [2]. Among them, the cultivation of *P. ginseng* in the forest is a bionic cultivation mode. The shape and the effective ingredients of *P. ginseng* planted in the forest are similar to wild ginseng, and the quality is superior comparatively [3]. In order to achieve medicinal characteristics with an efficacy comparable to that of wild ginseng, *P. ginseng* that has been planted in forests must grow continually in fixed plots for 15 to 20 years, or even longer [4]. *P. ginseng* soil-borne diseases become more serious with the cultivation years, reducing the *P. ginseng* survival rate [5].

Microbiota is a major component of agroecosystems [6]. As the most active component in the soil ecosystem, soil microorganisms can improve physical and chemical properties and regulate soil nutrition through their activities [7, 8]. Soil microbiotas directly participate in the circulation and metabolism of substances, and indirectly affect the growth and development of plants. Rhizosphere soil is a unique environment directly affected by plant roots and root exudates, and plays a key role in the interaction between plants and soil microorganisms [9]. Rhizospheric bacteria also act as a barrier against diseases and harmful substances found in soil [10]. As a result, the rhizospheric soil microbial community is an essential biological indicator of soil functioning [11, 12]. It is common for the structure and relative activity of rhizospheric microbiotas to vary from one plant species to another, as well as during stages of plant development and cultivation years [13, 14]. Recent research on the association between continuous cropping and microbial community showed that the diversity and composition of soil microbial community have alternated after continuously planted American ginseng (*Panax quinquefolius*) or *P. ginseng* (*Panax ginseng*) [15, 16]. In addition, different cropping years of *P. ginseng* will also alter the soil microbial community [17, 18].

The leaf litter of trees affects the forest soil nutrients, microbial community diversity, and composition structure. Alterations in the microbial populations of the soil might have an effect on diseases that are transmitted through the soil [19, 20]. Therefore, the selection of forest tree species is one of the keys to the success of cropping *P. ginseng* under the forest. The early investigation results showed that the occurrence probability of *P. ginseng* soil-borne disease in larch forests was generally lower than that in broad-leaved forests, and the survival rate of seedlings was higher [21, 22]. Therefore, it is necessary to systematically study and analyze whether the microbial diversity and community composition of *P. ginseng* rhizosphere soil under larch forests alter with *P. ginseng* cultivation and growth ages.

Owing to the next-generation sequencing technology allowing the capture of microbes without culturing the microorganisms, as well as the microbial community of difficult to cultivate or even unable to cultivate, microbial profiles analysis has become the main tool in plant science and agriculture [23, 24]. Therefore, this paper uses Illumina HiSeq high-throughput sequencing technology to analyze the 16S rRNA/ITS gene sequence of *P. ginseng* rhizosphere soil under larch forest, and study the diversity and community composition structure of rhizosphere microorganisms. At the same time, the accumulation level of soil-borne pathogens in the rhizosphere soil of *P. ginseng* after planting and different years was compared and analyzed, providing theoretical support for the selection of *P. ginseng* cropping sites and soil improvement under the forest.

## Materials and methods

### Collection of soil samples

*P. ginseng* rhizosphere soil samples and control soil samples (without cropping *P. ginseng*) were collected in July 2020 from the *P. ginseng* planting base (Beigou Village, Jindou Korean Manchu Autonomous Township, Tonghua County, Jilin Province) under larch forests that have been continuously planted for 2 (Y2), 5 (Y5) and 18 (Y18) years, respectively; following other researchers [25], as well as our previous study [18]. The soil samples without cropping *P. ginseng* were collected as controls, which were YCK2, YCK5, and YCK18, respectively. The cultivation mode of *P. ginseng* in the three sampling points is cultivated under forest. *P. ginseng* seeds are scattered in the forest, and there is no manual intervention during the growth of *P. ginseng*. During sample collection, the rainy weather was avoided and kept the soil natural dry. Removed the surface soil, dogged out *P. ginseng* roots, gently scraped the soil near the root surface (at least 5 g) with a cotton swab, transferred it into a marked sterilized bag, and stored it in an ice box for transportation to the laboratory. In this study, three samples were collected for each planting year for sequencing and subsequent analysis.

### DNA extraction, gene amplification, and high-throughput sequencing

We have used E.Z.N.A® Soil DNA Kit (OMEGA, U.S.) to extract the total DNA of microorganisms from the soil sample collected from the *P. ginseng* rhizosphere. NanoDrop 2000 ultraviolet–visible spectrophotometer (Thermo Scientific, U.S.) was used to detect the concentration and purification of the extracted DNA, and then 1% agarose gel electrophoresis was used for quality detection.

Using the V3-V4 region of the bacterial 16S rRNA gene as the target sequence, bacterial genome were amplified with a set of universal primers, for the positive strand as 338F(5'-ACTCCTACGGGAGGCAGCAG-3') and the negative strand as 806R(5'-GGACTACHVGGGTWTCTAAT-3') by following other researchers [26], and the amplified product was 468 bp. The Internally Transcribed Space (ITS) region as the fungal target sequence, was amplified with the universal primer set ITS1F (5'-CTTGGTCATTTAGAGAGGAAGTAA-3') / ITS2R (5'-GCTGCGTT CTTCATCGATGC-3) [27], and the amplified product was 300 bp. We have used ABI GeneAmp® 9700 thermocycler for DNA fragment amplification. We denatured DNA at 95 °C for 3 min with a single cycle; followed by denaturation at 95 °C for 30 s, annealed at 55 °C for 30 s with a total of 28 cycles, and extended at 72 °C for 45 s; and then allowed a final extension to the overall reaction at 72 °C for 10 min. The PCR reaction recipe is given in Table 1.

**Table 1:** The PCR reaction recipe for DNA fragment amplification

S. No	Reagent name	Concentration	Amount
1	FastPfu Buffer	5X	4 μL
2	dNTPs 2.5	2.5 μM	2 μL
3	Primer F	5 μM	0.8 μL
4	Primer R	5 μM	0.8 μL
5	FastPfu Polymerase	-	0.4 μL
6	DNA template	10 ng	1 μL
7	H_2_O	-	11 μL
The total amount of the reaction			20 μL

The amplified PCR product was screened using 1.5% agarose gel electrophoresis. The gel was purified using the AxyPrep DNA Gel Extraction Kit (Axygen Biosciences, U.S.). The gel-purified DNA was quantified using QuantiFluor™-ST (Promega, U.S.).

Following the standard procedure of Majorbio Bio-Pharm Technology Co., Ltd. (Shanghai, China https://www.majorbio.com/), the purified amplicons were equimolar merged, and paired-end sequencing was performed on the Illumina MiSeq platform (TruSeq™ DNA SamplePrep Kit, Illumina, U.S.). For bacterial community analysis, the clustering method used the USEARCH7-uparse algorithm; the OTU sequence similarity was 0.97, the species taxonomy database was silva138/16 s_bacteria, and the classification confidence was 0.7, has been used by other researchers also [28]. Similarly, for the Fungal community analysis, the clustering method used the USEARCH7-uparse algorithm; OTU sequence similarity: 0.97; Confidence: 0.7; species classification database used was ITS;unite8.0/its_fungi [29, 30]. The raw reads are submitted to NCBI's Sequence Read Archive (SRA) database; the accession numbers are PRJNA927237 and PRJNA928213.

### Sequence data analysis

The FastQ raw sequencing files were demultiplexed, the quality filter was applied using Trimmomatic [31]. The files were combined with FLASH [32]. We followed these standards: (i) reads were truncated at any point that received an average quality score of < 20 over a sliding 50-bp window, (ii)The primers were aligned exactly, permitted two nucleotide mismatches, and ambiguous reads were filtered out, and (iii) sequences with more than ten bp overlap were merged according to their overlap sequence. We used UCHIME (V4.2) [33], to identify and remove chimeric sequences, and used UPARSE (V7.1) [34] to cluster OTUs (Operational Taxonomic Units) sequences with a similarity threshold of 97%. Using the RDP Classifier [35]. The taxonomy of each 16S rRNA and ITS sequences were analyzed according to our previous research [18]. After OTU identification results, calculated α-diversity index from Mothur (V1.30.2). The Bray–Curtis distance algorithm was used to calculate the variance and β diversity. PCoA (Principal Co-ordinates Analysis) was investigated using ANOSIM; PERMANOVA was performed using the Bray–Curtis spacing algorithm with 999 permutations [36].

### Statistical analysis

This study used Student's *t*-test to compare alpha diversity indicators of different groups, and the statistical significance was set as * *p* < 0.05, ** *p* < 0.01, and *** *p* < 0.001. One-way ANOVA was used for the comparative study of multiple groups.

## Results

### Sequencing and depth analysis

In order to characterize the microbial community in the rhizosphere soil of *P. ginseng* under larch forests with different planting years, Illumina MiSeq sequenced eighteen samples. After filtering the sequencing data of eighteen *P. ginseng* rhizosphere soil samples, a total of 1,429,596 reads of bacterial 16S rRNA V3-V4 effective sequences, and 1,094,396 reads of fungal ITS effective sequences were obtained. The average length of bacterial and fungal sequences was 416 bp and 251 bp, respectively. Seven thousand ninety-four bacterial OTUs and 3486 fungal OTUs were obtained by clustering with 97% sequence similarity of high-quality sequences.

**Fig. 1:**
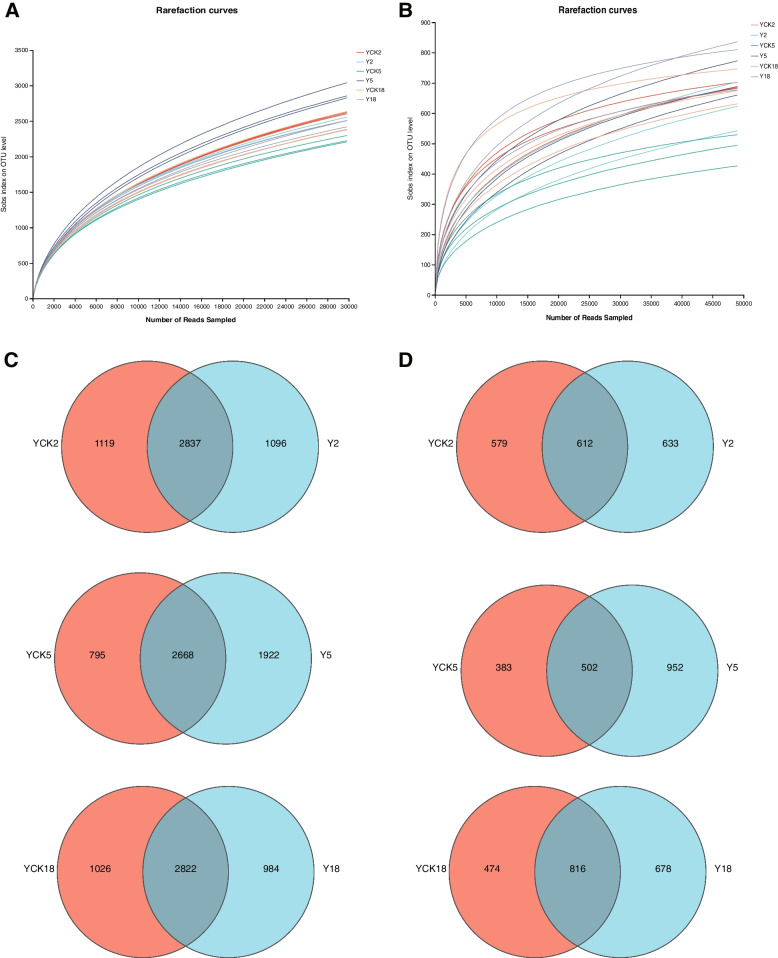
Rarefaction curves and Venn diagram of individual soil samples (**A**, **C**. bacteria, **B**, **D**. fungi). Details are provided in the text

Flattened the data set according to the minimum number of sequences of the samples, and constructed a rarefaction curve in this study. Based on sample OTU counts, rarefaction curves were created (Fig. 1A, B). It can be seen from the figure that the OTUs rarefaction curve of each sample tends to be flat, which indicates that the sequencing data is reasonable for estimating the diversity of bacteria and fungi of *P. ginseng* rhizosphere soil samples. Compared with bacterial rarefaction curves, fungal have a higher degree of variation in the shape of rarefaction curves.

From the Venn chart of OTU number (Fig. 1C, D), the proportion of unique OTU of *P. ginseng* rhizosphere bacteria in Y2, Y5, and Y18 was 21.69%, 35.69%, and 20.36%. The proportion of common OTU with non-planted *P. ginseng* soil bacteria was 56.16%, 49.55%, and 58.40%, respectively. It indicates the total OTU was significantly higher than the unique OTU. The proportion of unique OTU of *P. ginseng* rhizosphere fungi in Y2 and Y5 was 34.70% and 51.82%, while the proportion of common OTU with non-cultivated *P. ginseng* soil fungi was 33.55% and 27.33%, respectively. The unique OTU of Y2 and Y5 *P. ginseng* rhizosphere fungi was higher than the common OTU, while in Y18, the unique OTU of *P. ginseng* rhizosphere fungi (34.45%) was lower than the common OTU (41.46%).

### α- diversity analysis of microbial communities

**Fig. 2:**
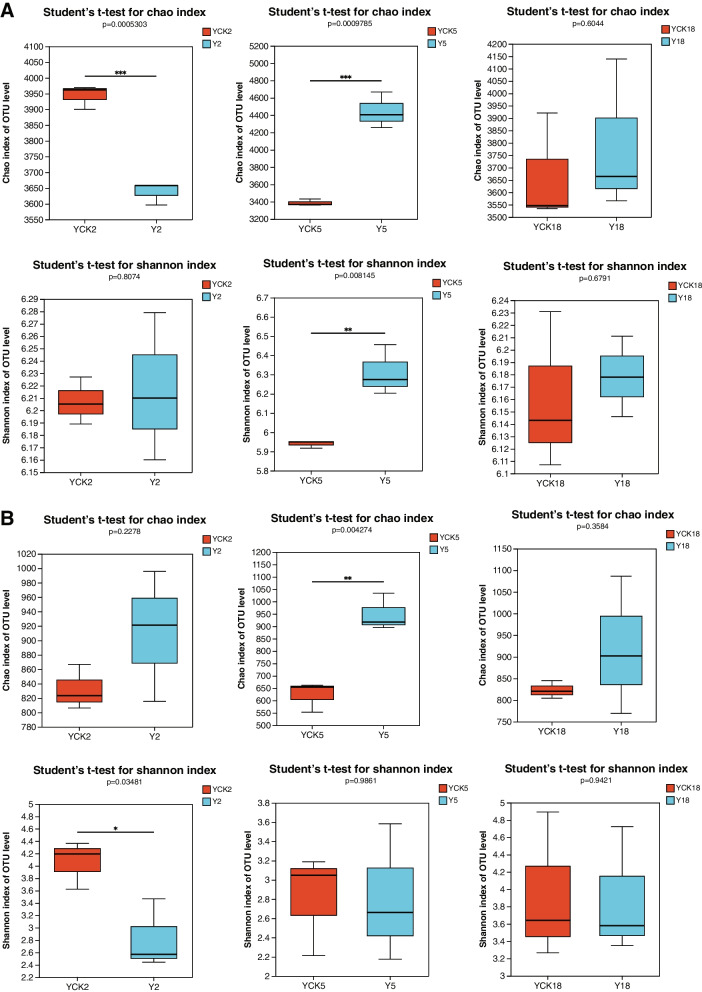
Estimation of the microbial community by α-diversity. **A**. α-diversity of bacterial communities. **B**. α-diversity of fungal communities. Estimation of α-diversity representing three biological replicates (* *p* < 0.05, ** *p* < 0.01, *** *p* < 0.001)

In order to estimate the differences of the microbial α-diversity, we removed the minimum number sequence from the data set of samples. The Chao richness index and the Shannon evenness index of OUT, were used to reflect the α diversity of microbial communities (Fig. 2). Compared with the soil samples without planting *P. ginseng*, the rhizosphere bacteria richness index Chao and fungi diversity index Shannon of rhizosphere soil planted *P. ginseng* for two years decreased significantly, while the bacteria diversity index Shannon and fungi richness index Chao had no significant difference. Compared with the control soil samples, the bacteria richness and diversity of *P. ginseng* rhizosphere soil planted for five years were significantly improved, and the fungi richness also increased, but the diversity of fungi did not change significantly. The richness and diversity of *P. ginseng* rhizosphere microorganisms (bacteria and fungi) after planting 18 years of *P. ginseng* have no significant difference compared with the control soil samples (Fig. 2). *P. ginseng* is a perennial plant that requires several years of consistent growth in the same site. We noticed that as the number of *P. ginseng* cropping years increased, there was a significant shift in the -diversity of the soil 'microbe's population. The above analysis results showed that after two years of continuous planting of *P. ginseng*, the rhizosphere soil microorganisms decreased, while after five years of continuous planting of *P. ginseng*, the rhizosphere soil microorganisms increased. The rhizosphere microbial community of long-term continuous cultivation of *P. ginseng* for 18 years had no significant alteration compared with the soil without planting *P. ginseng*.

### β- diversity analysis of microbial communities

**Fig. 3:**
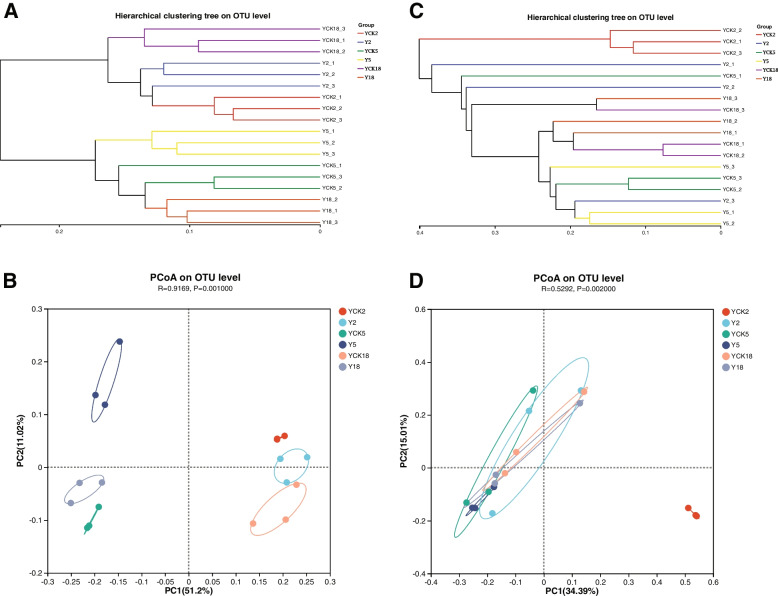
Comparative analysis at the OTU level using the hierarchical clustering tree of samples (**A**, bacteria; **C**, fungi) and PCoA (**B**, bacteria); **D**, fungi. The length of the branches in the tree diagram shows the distance between samples. The two samples are most likely to have a similar species composition when they are close to one another on the PCoA plot

We have estimated the β- diversity according to the phylogenetic level of OTUs of soil microbial communities. We used a Bray–Curtis distance matrix to assess the community composition across samples by estimating the abundance of reads after normalization and square root conversion. Hierarchical clustering was constructed based on Bray–Curtis dissimilarities. The hierarchically clustered pairwise Bray–Curtis dissimilarities of P. ginseng rhizosphere soil microbe OTUs revealed that the bacterial and fungal communities are fairly closely related, especially the bacteria of the same group samples are completely clustered (Fig. 3A, C).

In order to evaluate the general similarity of microbial community structures in samples, a PCoA analysis was performed; in conjunction with a PERMANOVA analysis, the PCoA results indicated that the community composition of bacteria (R2 = 0.9169, *p* = 0.001) and fungi (R2 = 0.5292, *p* = 0.002) differed significantly among the sample groups. PCoA analysis showed that the horizontal co-ordinate was the main co-ordinate component that caused the difference in microbial community composition in different soil samples. It is important to note that, concerning the OTUs, the variance contribution rates of PC1 were 51.20% and 34.39%, respectively, to the difference in the composition of the bacterial and fungal communities in the two samples (Fig. 3B, D).

**Fig. 4:**
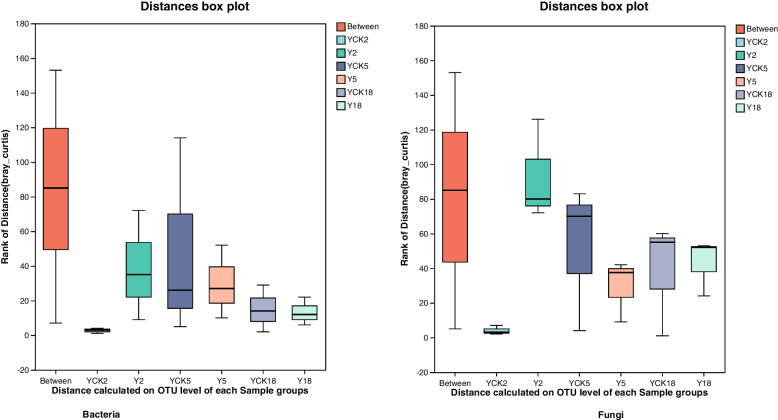
The ANOSIM analysis of similarities for i). Bacterial communities ii). Fungal communities

ANOSIM (|R|) was performed using a Bray–Curtis dissimilarity matrix with 999 permutations to show whether the groups' microbial community structures were significantly different. The "ANOSIM" analysis charts are provided below for bacterial and fungal communities in Fig. 4.

### Composition and structure of the bacterial community

**Fig. 5:**
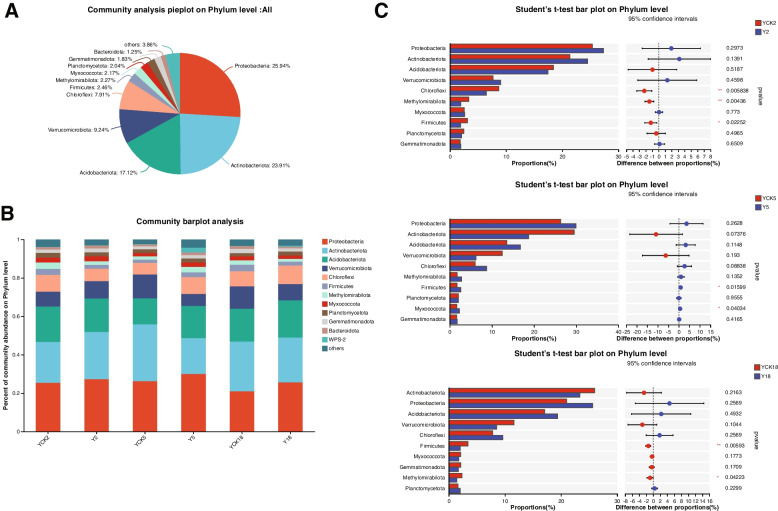
The composition and organization of the bacterial colony. **A**. Pieplot for phylum-level investigation of bacterial communities. **B**. The relative abundances of the various bacterial phyla. **C**. A phylum-level assessment of the presence of bacteria in soil samples taken from planted and unplanted P. ginseng. For every rhizospheric soil sample taken, there were triple biological duplicates. (* *p* < 0.05, ** *p* < 0.01, *** *p* < 0.001)

The rhizosphere bacteria of P. ginseng under larch forest identified 38 phyla, 118 classes, 283 orders, 445 families, 798 genera, 1803 species, and 7094 OTU based on 97% species similarity. The phyla Proteobacteria (25.94%), Actinobacteriota (23.91%), Acidobacteriota (17.12%), Verrucomicrobiota (9.24%), and Chloroflexi (7.91%) were the richest in all the eighteen *P. ginseng* rhizosphere soil samples (Fig. 5A). We investigated the leading bacterial phyla and perceived distinct bacterial communities in the soil samples cropping *P. ginseng*. The soil microbial community structure differed in diverse years of continuous planting of *P. ginseng* (Fig. 5B). The first ten phyla were rated using ANOVA to test the impact of different cultivation years (YCK2 vs. Y2, YCK5 vs. Y5, YCK18 vs. Y18) on their relative abundance in percent values (Fig. 5C). Verrucomicrobiota, Chloroflexi, Firmicutes, Methylomirabilota, and Myxococcota in the top ten relatively abundant bacterial phyla were significantly different among distinct samples. The significant difference between the bacterial communities of planted and non-planted *P. ginseng* soil was further assessed by 'Student's t-test (Fig. 5C). Compared with no planted *P. ginseng* soil samples, the relative abundance of dominant bacteria Chloroflexi (YCK2 8.711%, Y2 6.495%), Methylomirabilota (YCK2 3.367%, Y2 1.934%), and Firmicutes (YCK2 3.130%, Y2 1.916%) were significantly decreased in the rhizosphere soil continuous planting P. P. ginseng for two years (p < 0.05). However, Firmicutes (YCK5 1.698%, Y5 2.583%), and Myxococcota (YCK5 1.675%, Y5 2.283%) were significantly increased in continuous cultivated *P. ginseng* soil for five years. After 18 years of *P. ginseng* continuous cultivation in larch forest, the relative abundance of Firmicutes (YCK18 3.430%, Y18 2.025%), and Methylomirabilota (YCK18 2.342%, Y18 1.435%) decreased significantly in Y18 as compared to that in YCK18 (Fig. 5C).

### Composition and structure of the fungal community

**Fig. 6:**
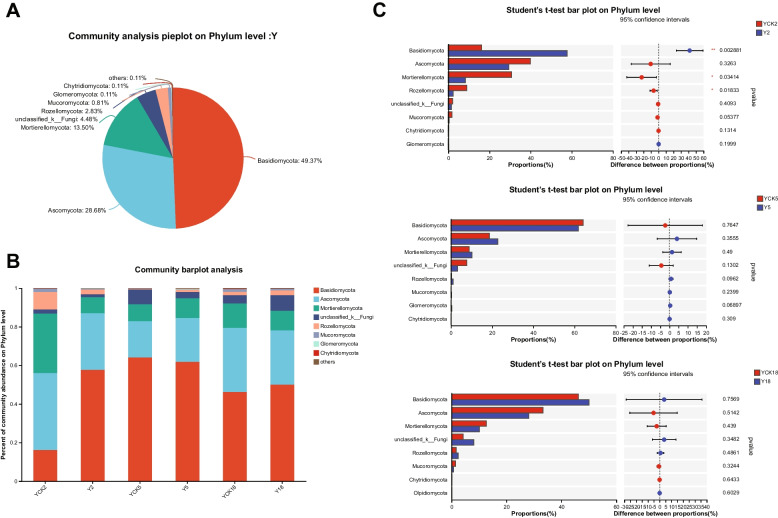
Fungal community composition and structure. **A**. A phylum-level pie chart for analyzing fungal communities. **B**. The diversity of fungal phyla across collections and their relative abundances. **C**. Comparison of fungal abundance in the planted and non-planted *P. ginseng* soil samples at the phylum level. Three replicates were used for each rhizospheric soil sample (* *p* < 0.05, ** *p* < 0.01, *** *p* < 0.001)

From fungal ITS sequences of *P. ginseng* rhizosphere soil, 15 phyla, 63 classes, 156 orders, 339 families, 709 genera, 1158 species, and 3486 OTU were identified. Fungal OTUs predominantly comprised of phyla Basidiomycota (49.37%), Ascomycota (28.68%), Mortierellomycota (13.50%), Rozellomycota (2.83%), Mucoromycota (0.81%), Chytridiomycota (0.11%) and Glomeromycota (0.11%) (Fig. 6A). Basidiomycota showed the highest abundance in the soil samples of larch forest while cultivating *P. ginseng* (Fig. 6A). By comparing the fungal communities in non-planting P. ginseng soil and three distinct years of P. ginseng cultivation, we could determine the dominating fungal phyla (Fig. 6B). The relative abundance of Basidiomycota and Rozellomycota varied significantly (*p* < 0.05) on different soil samples among nearly all identified fungal phyla. To determine whether there was a significant difference between the fungal communities in the non-cropping *P. ginseng* soils, the Student's t-test was conducted. (YCK2, YCK5, YCK18) and continuous planting of *P. ginseng* soil samples (Y2, Y5, Y18). In the second year of *P. ginseng* cultivation, there was an increase in the relative abundances of Basidiomycota in Y2 (57.720%) compared to those in the YCK2 soil sample, which was 16.210% for the phyla. While the relative abundance of Mortierellomycota (YCK2 30.780%, Y2 8.264%), and Rozellomycota (YCK2 9.011%, Y2 2.403%) decreased remarkably in Y2 compared to that in YCK2 (Fig. 6C). After continuous planting *of P. ginseng* for five or eighteen years in the larch forest field, the relative abundances of fungal phyla did not vary significantly in Y5 and Y18 as compared to that in YCK5 and YCK18, respectively (Fig. 6C).

### The multi-group comparative analysis

**Fig. 7:**
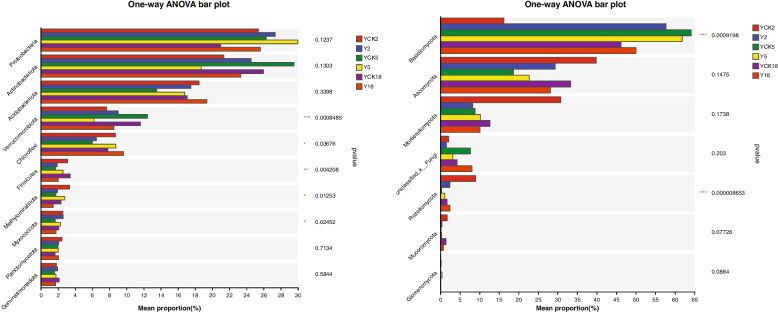
The multi-group comparative analysis; i). Bacterial communities ii). Fungal communities

The multi-group comparative analysis on 3 samples Y2, Y5 and Y18 has been conducted for bacterial and fungal communities. The results are shown in Fig. 7.

### Accumulation of pathogenic fungi with cropping *P. ginseng*

**Fig. 8:**
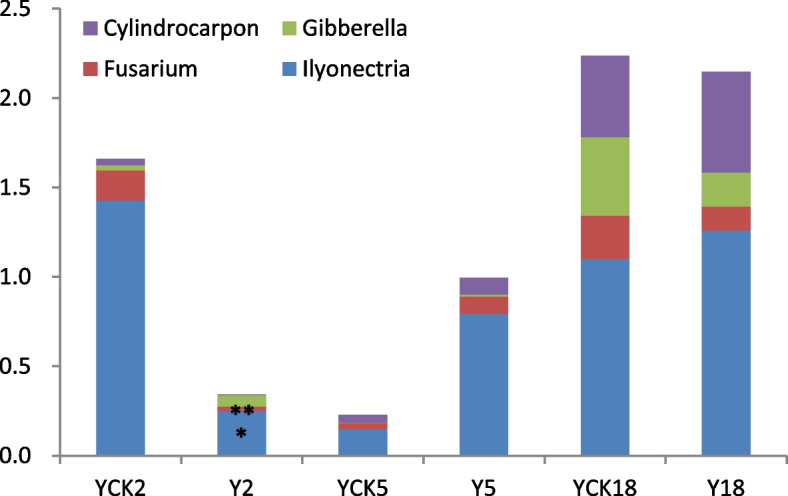
The relative abundance of different pathogenic fungi in soil samples. Three replicates were used for each sample (* *p* < 0.05, ** *p* < 0.01, *** *p* < 0.001)

*P. ginseng* is a perennial plant, which needs to be planted continuously for many years. With the increase in the number of *P. ginseng* cropping years, the accumulation level of pathogenic fungi in the soil rises, and the soil-borne disease of *P. ginseng* becomes progressively serious [18]. An early investigation found that the incidence of *P. ginseng* diseases under the larch forest was low, and the disease index was small. Therefore, it is speculated that with the *P. ginseng* cropping and increase of planting years, the accumulation level of *P. ginseng* related pathogenic fungi in the soil under larch forest is insignificant. *Fusarium* is a common pathogenic fungi that causes soil-borne diseases in plants, including *P. ginseng* [37]. *Gibberella*, the perfect stage of *Fusarium*, was also detected in the eighteen soil samples [38]. In addition to *P. ginseng* root rot, another major soil-borne disease is *P. ginseng* rust rot, which is caused by *Cylindrocarpon* and/or *Ilyonectria* [39]. Accordingly, we analyzed the changes in the abundances of these four pathogenic species with cropping *P. ginseng* under a larch forest. We found that four pathogenic species (*Fusarium*, *Gibberella*, *Cylindrocarpon*, and *Ilyonectria*) accumulated differently in *P. ginseng* rhizosphere soil samples of different plantation years (Fig. 8).

**Fig. 9:**
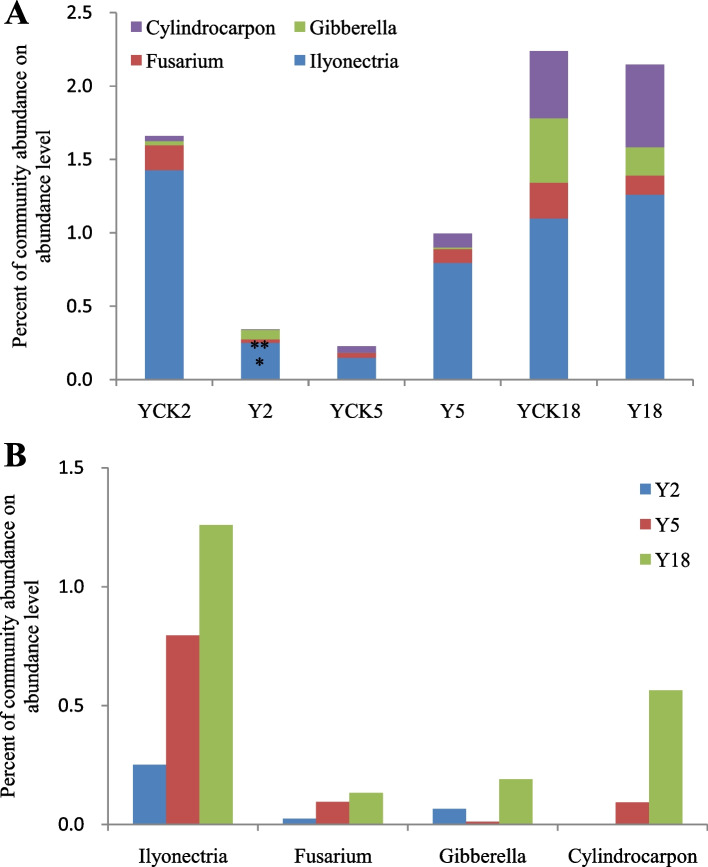
The comparative results of four pathogens among three samples, Y2, Y5, and Y18, reveal the changes with the extension of planting time. **A**. Percent community abundance of the four fungal pathogens **B**. Difference in the fungal pathogens distribution with passage of time

The results of this study showed that the total number of four kinds of pathogenic fungi in the rhizosphere soil of *P. ginseng* planted for two years (Y2) was significantly reduced compared with that in the larch forest soil without cropping *P. ginseng* (YCK2). The content of *Ilyonectria* (YCK2 1.43%, Y2 0.25%, *p* < 0.05) and *Fusarium* (YCK2 0.17%, Y2 0.03%, *p* < 0.01) in Y2 decreased significantly compared with that of YCK2, as shown in Fig. 9A&B. Compared with the larch forest soil samples (YCK5), the sum of four kinds of pathogens in the rhizosphere soil of *P. ginseng* cultivated for five years (Y5) showed an increasing trend. Although the total number of pathogenic fungi in Y5 increased significantly, four pathogenic species' accumulation level did not vary significantly compared with YCK5. However, the total amount of four pathogens in the rhizosphere soil of *P. ginseng* planted continuously for 18 years (Y18) is nearly the same as that in the larch forest soil without planting *P. ginseng* (YCK18). And the accumulation level of these four pathogenic fungi in Y18 did not increase significantly with continuously planted *P. ginseng* for 18 years. In short, the abundance of these four types of *P. ginseng* pathogens was less. And the accumulation level of pathogens (*Fusarium*, *Gibberella*, *Cylindrocarpon*, and *Ilyonectria*) did not increase significantly compared with the control samples with the *P. ginseng* cropping under larch forest and the increase of cultivation years.

## Discussion

*P. ginseng* is a perennial, slow-growing plant primarily used in Chinese herbal remedies. During the long-term natural and artificial selection, three cultivation modes have been formed, including cultivated *P. ginseng* under the forest, cultivated *P. ginseng* in deforestation, and cultivation of *P. ginseng* in the farmland. Cultivated *P. ginseng* under the forest, namely forest *P. ginseng*, is developed by sowing *P. ginseng* seeds into the natural environment, allowing them to grow without any artificial interference or management, and its growth usually exceeds ten years [40]. When grown in a forest, *P. ginseng* is more similar to wild ginseng than when it is grown in a farm [41]. Several factors such as climate change and soil properties affectplant's survival [42]. The soil-borne diseases of *P. ginseng* are aggravated with the increase in cultivation years, decreasing the persistence rate of *P. ginseng*. An early investigation found that the growth status and survival rate of *P. ginseng* in larch forests were generally better than those in broad-leaved forests [43, 44]. Tree species not only affect the shade degree of the forest, but also influence the soil microbial community composition [45]. These microbes play an important role in the completion of nutrients cycles. Therefore, the selection of forest tree species is one of the important factors for the success of cropping *P. ginseng* under the forest.

Microbial communities can be employed as sensitive biological markers of soil health and function, and they play an important role in sustaining soil ecosystem stability and health [46, 47]. Variations in microbial communities may have adverse effects on soil quality and plant health. Previous research has found that *P. ginseng* cultivation, particularly on diverse lands and for varying lengths of time, affects the richness and organization of the *P. ginseng* rhizosphere microbial population [48, 49]. In addition, several other factors can affect root biomass and morphology [50]. This study found that the richness and evenness of soil microbial communities both fungi and bacteria in larch forests, transformed substantially with the different planting years of *P. ginseng*. Compared with the larch forest soil without cropping *P. ginseng*, the microbial community of *P. ginseng* rhizosphere soil changed significantly after planting *P. ginseng* for two or five years, while the microbial community of *P. ginseng* rhizosphere soil did not alteration after cultivating *P. ginseng* for 18 years (Fig. 2). The microbial communities in rhizosphere soil of the same annual *P. ginseng* under larch forest are relatively close in the hierarchical clustering of pairwise Bray–Curtis dissimilarities, especially since the bacteria in the same group of samples are completely clustered (Fig. 3A, C). PCoA results showed that the community composition of bacteria (R2 = 0.9169,* p* = 0.001) and fungi (R2 = 0.5292, *p* = 0.002) in different samples was significantly different (Fig. 3B, D). The results showed that the microbial composition of *P. ginseng* rhizosphere soil varied significantly with the cultivation years. Other researchers studied microbial communities in different environments [51].

The rhizosphere microbial community may be influenced by the soil's characteristics and crop variety [52, 53]. The key factor for this change is planting years [54]. The plant's developmental stage is a major factor in determining the microbiome composition [55]. Root exudates at various phases of development may be responsible for this phenomenon [56, 57], which is responsible for this change of the rhizosphere microbiome community, during plant growth [52, 58]. The bacterial phyla Proteobacteria, Actinobacteriota, Acidobacteriota, Verrucomicrobiota, and Chloroflexi, and the fungal phyla Basidiomycota, Ascomycota, and Mortierellomycota were the most abundant during *P. ginseng* cultivation under larch forest in the current study, as we have indicated in Figs. 5A and 6A. Our findings are in accordance with other researchers' reports on farmland *P. ginseng* [18] and other crops (cotton) [59], soybean [60], and peanut [61]. Habitat specialization is important in species richness [62, 63]. These microbial phyla were abundant in *P. ginseng's* soil ecosystem. Proteobacteria was most significantly enriched in 18 larch forest soils (Fig. 5A). Proteobacteria, related to eutrophic soil [64, 65], plays an essential role in the global nitrogen, sulfur, iron, and carbon cycles [66, 67]. The second most prevalent group, Actinobacteriota, takes part in the decomposition of soil organic matter and the global carbon cycle [68, 69]. Actinobacteriota abundance fluctuated with the *P. ginseng* culture years, demonstrating that the *P. ginseng* cropping had a significant impact on the Actinobacteriota population. As a result, individuals of both Proteobacteria and Actinobacteriota may play a part in maintaining microbial homeostasis in soil used for continuous cropping. The soil nutrients have been influenced by several types of microbes [70].

The allelochemicals found in *P. ginseng* have been shown to cause disruption to the delicate equilibrium of the microbiome [71], reduce beneficial fungi, and increase pathogenic fungi in the rhizosphere soil of *P. ginseng* [72]. The preliminary investigation found that *P. ginseng* under larch forest grew well, and the incidence of *P. ginseng* disease was low. Therefore, it is speculated that with the *P. ginseng* cropping and increase of cultivation years, the accumulation level of *P. ginseng* related pathogens in the soil under larch forest is insignificant. *P. ginseng* root rot caused by *Fusarium*, and *P. ginseng* rust rot caused by *Cylindrocarpon* and *Ilyonectria* are important soil-borne diseases in *P. ginseng* cultivation [73, 74]. Furthermore, *Gibberella*, a perfect stage for *Fusarium* [38], is also an important soil-borne pathogen. The analysis of pathogenic species found that the accumulation level of *Fusarium*, *Gibberella*, *Cylindrocarpon*, and *Ilyonectria* in the rhizosphere soil of different age *P. ginseng* under the larch forest was different (Fig. 8). The research results indicated that the summation of four kinds of pathogenic fungi in Y2 was significantly decreased compared with that in YCK2. The amount of *Ilyonectria* and *Fusarium* in Y2 reduced significantly compared with that of YCK2. Compared with YCK5, the total amount of four pathogens in Y5 showed an increasing trend, but the accumulation level of four pathogenic species did not variation significantly. Compared with YCK18, the accumulation level of four pathogenic fungi in Y18 did not increase significantly with continuously cropping *P. ginseng* for 18 years (Fig. 8).

## Conclusions

The results of this study showed that the pathogens (*Fusarium*, *Gibberella*, *Cylindrocarpon*, and *Ilyonectria*) in the soil of the larch forest changed little or not significantly with the *P. ginseng* planting and the increase of cultivation years. It suggested that the soil function of the larch forest was good. This study provides a theoretical basis for the land selection and soil improvement of cultivating P. ginseng under forest.

### Supplementary Information

**Additional file 1: Table S1. **The software’s used in this study. 

## Data Availability

The dataset(s) supporting the conclusions of this article are available online at NCBI Sequence Read Archive (SRA) database (Accession Number: PRJNA927237 and PRJNA928213).
