# Supplementary material for: Comparative analysis of microbial community structure in different times of Panax ginseng Rhizosphere microbiome and soil properties under larch forest
Source: BMC Genom Data. 2023 Sep 14;24:51. doi: 10.1186/s12863-023-01154-1 (PMC10500862; doi:10.1186/s12863-023-01154-1)
Supplement: Supplementary file 1 — Additional file 1: Table S1. The software’s used in this study. [file 12863_2023_1154_MOESM1_ESM.docx]

**Supplementary information**

Table_S1. The software’s used in this study.

| Software/database | VERSION | Analysis software/database usage | Analysis software/database link) |
| --- | --- | --- | --- |
| Flash | 1.2.11 | pair-end | https://ccb.jhu.edu/software/FLASH/index.shtml |
| Qiime | 1.9.1 |  | http://qiime.org/install/index.html |
| Uparse | 7.0.1090 | OTU | http://www.drive5.com/uparse/ |
| RDP Classifier | 2.11 |  | https://sourceforge.net/projects/rdp-classifier/ |
| Usearch | 7 | OTU | http://www.drive5.com/usearch/ |
| Mothur | 1.30.2 | alpha | https://www.mothur.org/wiki/Download_mothur |
| PICRUSt | 1.1.0 | 16S, KEGG、COG、Pfam | http://picrust.github.io/picrust/ |
| Mega | 7 |  | https://www.megasoftware.net/ |
| SILVA | 138 | rRNA | https://www.arb-silva.de/ |
| UNITE | 8 | ITS | https://unite.ut.ee/ |
| RDP | 11.5 | rRNA | http://rdp.cme.msu.edu/ |
| GreenGenes | 135 | rRNA | http://greengenes.secondgenome.com/ |
| FunGene | 9.6 |  | http://www.fungene-db.fr/ |
| MaarjAM | 81 | 18SrRNA | https://www.maarjam.botany.ut.ee/ |
| HPB | -- | 16SrRNA | https://www.cerl.org/resources/hpb/content |
| PR2 | 1 | 18SrRNA | https://github.com/vaulot/pr2_database https://figshare.com/articles/PR2_rRNA_gene_database/3803709 |
| Funguild | 1 |  | http://www.funguild.org/ |
| Tax4fun | 0.3.1 | Tax4Fun | http://tax4fun.gobics.de/ |
| MAFFT | 7.2 |  | https://mafft.cbrc.jp/alignment/software/ |
| IQ-TREE | 1.6.8 |  | http://www.iqtree.org/ |
| Fastp | 0.19.6 |  | https://github.com/OpenGene/fastp |
| PICRUSt2 | 2.2.0 | KEGG orthologys (KO) 、EC、COG, MetaCyc | https://github.com/picrust/picrust2/ |
